# Dynamic physiological and transcriptomic changes reveal memory effects of salt stress in maize

**DOI:** 10.1186/s12864-023-09845-w

**Published:** 2023-12-01

**Authors:** Zhiying Zhu, Yan Dai, Guangrun Yu, Xin Zhang, Qi Chen, Xiaobing Kou, Eid M. Mehareb, Ghulam Raza, Baohong Zhang, Baohua Wang, Kai Wang, Jinlei Han

**Affiliations:** 1 School of Life Sciences, Nantong University, Nantong, 226019 China; 2 College of Agriculture, Fujian Agriculture and Forestry University, Fuzhou, 350002 China; 3 Sugar Crops Research Institute, Agricultural Research Center, Giza, 12619 Egypt; 4 National Institute for Biotechnology and Genetic Engineering, College Pakistan Institute of Engineering and Applied Sciences (NIBGE-C, PIEAS), Faisalabad, 38000 Pakistan; 5 Department of Biology, East Carolina University, Greenville, NC 27858 USA

**Keywords:** Stress memory, Salt stress, Regulatory network, Memory factor, Maize

## Abstract

**Background:**

Pre-exposing plants to abiotic stresses can induce stress memory, which is crucial for adapting to subsequent stress exposure. Although numerous genes involved in salt stress response have been identified, the understanding of memory responses to salt stress remains limited.

**Results:**

In this study, we conducted physiological and transcriptional assays on maize plants subjected to recurrent salt stress to characterize salt stress memory. During the second exposure to salt stress, the plants exhibited enhanced salt resistance, as evidenced by increased proline content and higher POD and SOD activity, along with decreased MDA content, indicative of physiological memory behavior. Transcriptional analysis revealed fewer differentially expressed genes and variations in response processes during the second exposure compared to the first, indicative of transcriptional memory behavior. A total of 2,213 salt stress memory genes (SMGs) were identified and categorized into four response patterns. The most prominent group of SMGs consisted of genes with elevated expression during the first exposure to salt stress but reduced expression after recurrent exposure to salt stress, or vice versa ([+ / −] or [− / +]), indicating that a revised response is a crucial process in plant stress memory. Furthermore, nine transcription factors (TFs) (WRKY40, WRKY46, WRKY53, WRKY18, WRKY33, WRKY70, MYB15, KNAT7, and WRKY54) were identified as crucial factors related to salt stress memory. These TFs regulate over 53% of SMGs, underscoring their potential significance in salt stress memory.

**Conclusions:**

Our study demonstrates that maize can develop salt stress memory, and the genes identified here will aid in the genetic improvement of maize and other crops.

**Supplementary Information:**

The online version contains supplementary material available at 10.1186/s12864-023-09845-w.

## Background

Soil salinization, typically caused by the accumulation of NaCl, is widespread abiotic stress that has deleterious impacts on plant growth and distribution. The adverse effects of salt stress can be attributed to two primary causes as follows [1–3]: On one hand, there is osmotic stress, which results in reduced root water absorption, stomatal closure, and inhibition of cell expansion. On the other hand, there is ionic toxicity, which causes oxidative damage and excessive generation of reactive oxygen species (ROS). Excessive ROS can damage membranes, disturb cellular metabolism, and accelerate plant senescence and death. Therefore, comprehending the underlying mechanisms governing plant responses to salt stress and the formulation of efficacious strategies for enhancing salt tolerance emerge as pivotal approaches to mitigating the loss of arable land due to soil salinization.

Plants have evolved sophisticated mechanisms to respond and adapt to abiotic stresses, which involve complex networks of numerous genes. A pivotal aspect of stress response is the regulation of functional genes by transcription factors (TFs), including various members of the NAC, WRKY, MYB, and bHLH families [2, 4–6], which activate protective mechanisms enabling plants to cope with abiotic stress. Notably, recent studies have demonstrated that plants can memorize their previous exposure to abiotic stress at physiological and transcriptional levels [7–9], leading to enhanced tolerance or resistance upon subsequent stress encounters, which is called stress memory [10]. For example, dehydration stress memory has been observed in several plant species, including *Arabidopsis* [11, 12], maize [13, 14], and switchgrass [7]. Plants that have experienced dehydration/recovery cycles exhibit improved performance, including an increased ability to maintain leaf relative water content when subsequently exposed to dehydration stress. By using transcriptome deep sequencing (RNA-seq), genes associated with dehydration stress memory have been identified [7, 13]. These genes exhibited significantly divergent transcript responses between the initial and subsequent stress events, and they can be classified into four categories as follows: (1) genes that are up-regulated during the initial stress event and exhibit elevated expression during the subsequent stress event (designated as [+ / +]), (2) genes that are down-regulated during the initial stress event and display reduced expression during the subsequent stress event ([-/-]), (3) genes that are up-regulated during the initial stress event but experience reduced expression during the subsequent stress event ([+/-]) and (4) genes that exhibit the opposite pattern ([-/ +]). Stress memory results in fewer changes in gene expression during subsequent exposure to the same type of stress [15, 16], suggesting a reduction in the disturbance of gene expression. Interestingly, studies comparing *Arabidopsis*, maize, and switchgrass have revealed the existence of both conserved and species-specific mechanisms that regulate plant responses to recurring dehydration stress [7], highlighting the intricate regulatory landscape of stress memory across plant species. Meanwhile, there is emerging evidence to suggest that epigenetic mechanisms, including DNA methylation and histone modifications such as glycosylation, acetylation, ubiquitination, and methylation alterations, serve as pivotal contributors to the establishment of environmental stress memory [17–20].

Maize (*Zea mays*), a globally significant cereal crop, plays a pivotal role in providing food, feed and bioenergy. However, its susceptibility to salt stress, particularly during the seedling stage, imposes considerable constraints on its growth, development, yield, and quality [21, 22]. To mitigate this challenge, it becomes imperious to: (1) elucidate the molecular mechanisms underlying salt tolerance in maize, (2) identify novel stress-resistant genes, and (3) breed new cultivars with enhanced resilience to salt stress. Stress memory enables plants to mount vigorous and rapid responses upon subsequent encounters with such stresses, consequently facilitating their recuperation. Therefore, the salt stress resistance and productivity of maize can be improved by inducing stress memory. However, our current understanding of salt stress memory and the underlying molecular mechanisms in maize remains limited.

In this study, we investigated the phenomenon of salt stress memory in maize using a precisely controlled liquid culture-based experimental framework. Maize plants were subjected to recurrent salt stress (200 mM NaCl for 0 h, 3 h, and 48 h), and their phenotypic, physiological, and transcriptomic responses to salt stress were evaluated. The results revealed that salt stress memory was established in maize. Protective components, such as proline, and processes, notably photosynthesis, were found to play an important role in the development of salt stress memory. Furthermore, we revealed the significance of TFs in initiating the salt stress memory in maize and identified nine pivotal TFs associated with stress memory. These results provide valuable insights into the mechanisms underlying salt stress memory in maize and may help to design genetic improvement strategies aimed at enhancing the salt stress resistance and productivity of maize.

## Results

### Phenotypic and physiological analyses indicated that maize displays salt stress memory

**Fig. 1:**
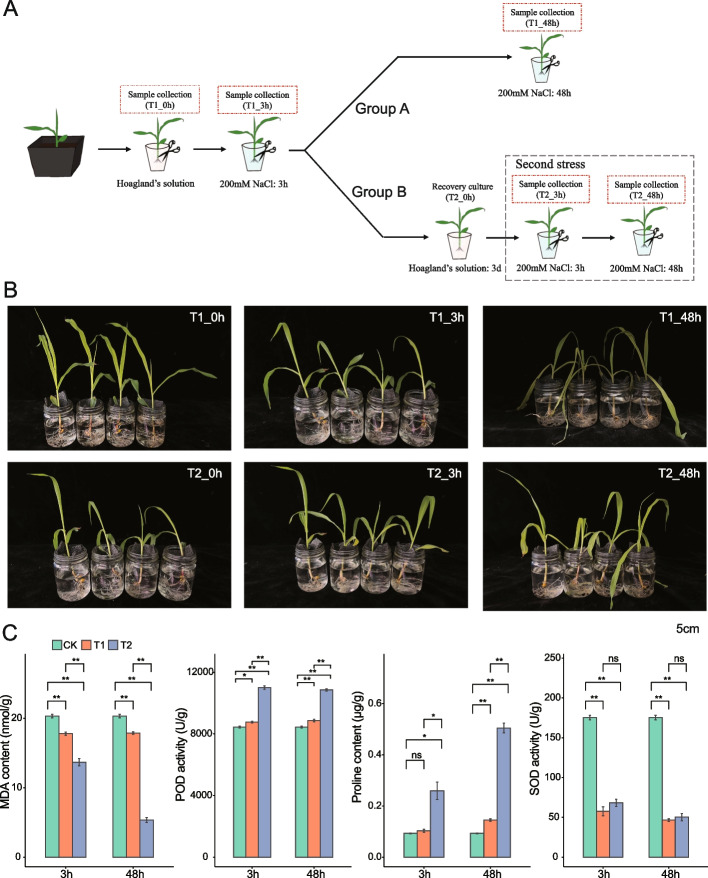
Morphological and physiological characteristics of maize in response to salt stress. **A** Experimental design of salt stress exposure. Plants were exposed to salt stress, and samples were collected at 0 h, 3 h and 48 h. T1_0h, Control; T1_3h and T1_48h, plants exposed to 200 mM NaCl for 3 h and 48 h, respectively; T2_0h, plants pretreated with 200 mM NaCl for 3 h, followed by 3 days of recovery culture; T2_3h and T2_48h, pretreated plants exposed to 200 mM NaCl for 3 h and 48 h, respectively. **B** Phenotypes of seedlings under salt stress. Photos were taken at different time points after salt treatment. **C** Effects of salt stress on the physiological parameters of maize. Data are presented as the mean ± standard error (SE) (*n* = 3). Student’s t-test was used for statistical analysis (* *P* < 0.05, ** *P* < 0.01, ns means no significant difference). CK, Control; T1, first salt stress event; T2, second salt stress event

A salt stress experiment was conducted to investigate the presence of salt stress memory in maize plants (Fig. 1A). Briefly, maize seedlings were cultivated in pots for one week and subsequently transferred to a hydroponic culture using Hoagland’s liquid medium for five days. Subsequently, the plants were exposed to 200 mM NaCl for 3 h, and then divided into two distinct experimental groups: Group A and Group B. Plants in Group A were subjected to an additional 45 h exposure to 200 mM NaCl (a total of 48 h). Plants in Group B were transferred to Hoagland’s liquid medium for three days of recovery culture before the second exposure to salt stress. Plants subjected to salt stress for 0 h, 3 h, and 48 h during both the first and second stress events were photographed and observed phenotypically (Fig. 1B). After 48 h of salt stress, it was evident that plants in Group A exhibited a more severe stress phenotype compared to those in Group B, characterized by pronounced leaf wilting, suggesting the development of salt stress memory following the initial salt stress treatment.

To validate these findings, root samples were collected for the measurement of physiological parameters, including proline and malondialdehyde (MDA) content, and the activity of superoxide dismutase (SOD) and peroxidase (POD) (Fig. 1C). Compared with non-saline conditions, exposure to salt stress for 3 h and 48 h induced a significant increase in proline content and POD activity, while decreasing MDA content. Notably, these changes were more pronounced in plants subjected to pretreatment. Moreover, salt stress led to a substantial reduction in SOD activity in both un-pretreated and pretreated plants; however, SOD activity trended to be higher in pretreated plants than in un-pretreated plants. Overall, these results indicated that a 3 h salt pretreatment significantly enhances the salt tolerance ability of maize plants, affirming the presence of salt stress memory in salt-pretreated plants.

### RNA-seq analysis revealed distinct transcriptional responses to repeated salt stress events

**Fig. 2:**
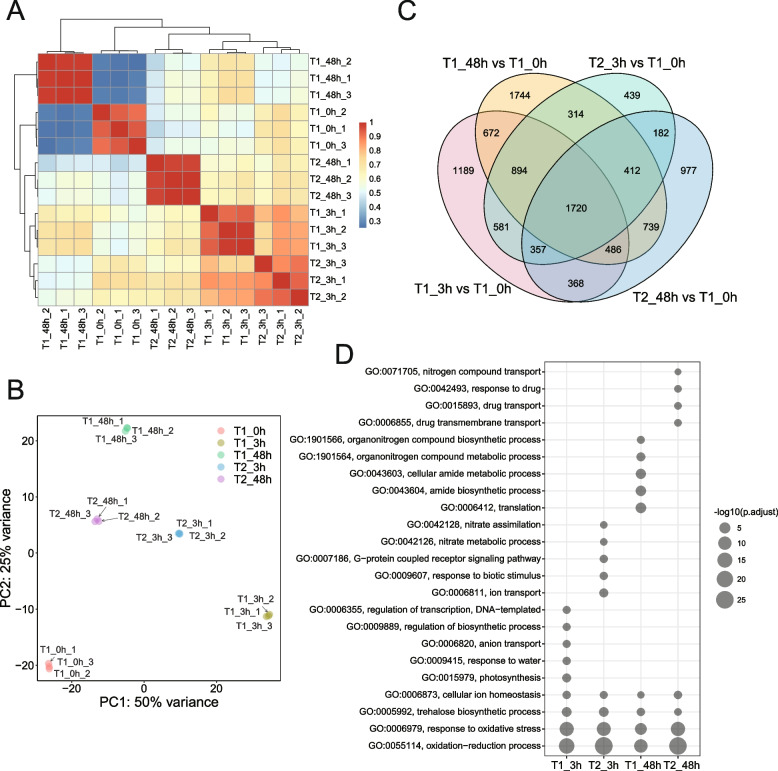
Differential expression analysis of maize in response to salt stress. **A** Heatmap displaying the Pearson correlation coefficient between samples based on RNA-seq data. **B** PCA plot of RNA-seq data. Each dot represents one sample. **C** Venn diagrams showing the overlap of DEGs between salt-stressed samples (T1_3h, T1_48h, T2_3h, and T2_48h) compared to the control samples (T1_0h). **D** GO analysis showing the enriched biological processes of each DEGs set

To examine the impact of recurrent exposure to salt stress at the transcriptomic level, RNA-seq was performed using root samples collected at 0 h, 3 h, and 48 h of salt treatment (designated as T1_0h, T1_3h, and T1_48h, respectively), and 3 h and 48 h of a subsequent exposure to salt stress (designated as T2_3h and T2_48h, respectively) (Fig. 1A). A total of 579.5 million paired-end reads were obtained, with an average of 38.6 million reads per sample (Table S1). These reads were mapped to the maize reference genome, with an average mapping rate of 72.9%, consistent with what was previously reported [23]. Pearson correlation coefficients and Principal component analysis (PCA) demonstrated a high similarity among biological replicates (Fig. 2A and B), suggesting the high quality and reproducibility of the RNA-seq data.

A total of 11,074 differentially expressed genes (DEGs) (fold change ≥ 2 and adjusted *P*-value ≤ 0.05) were identified in the T1_3h, T1_48h, T2_3h, and T2_48h groups when compared to the T1_0h control group (Fig. 2C, Table S2). Notably, the number of DEGs increased with the increasing duration of stress exposure during both the first and second stress events (T1_3h: 6,267 DEGs; T1_48h: 6,981 DEGs; T2_3h: 4,899 DEGs; T2_48h: 5,241 DEGs), indicating the activation or suppression of a greater number of genes as stress duration prolonged. Intriguingly, we observed that the number of DEGs in the T2_3h and T2_48h plants decreased by 21.8% and 24.9% when compared with the T1_3h and T1_48h plants, respectively. The greater number of DEGs identified at the first exposure to salt stress may be responsible for the more severe phenotypic changes (Fig. 1B). To further illustrate the functional roles of these DEGs, we performed Gene Ontology (GO) enrichment analysis for each of the four sets of DEGs (Fig. 2D, Table S3). This analysis revealed that they shared several enriched GO terms involved in the oxidation–reduction process, response to oxidative stress, trehalose biosynthetic process, and cellular ion homeostasis (Fig. 2D), which are critical processes for plant stress adaptation. However, there were also substantial differences in enriched GO terms between the first and second salt stress events. GO terms related to photosynthesis, water response, anion transport, and transcription regulation were predominantly enriched in the T1_3h samples, whereas GO terms related to ion transport, G-protein coupled receptor signalling pathway, and nitrate assimilation were enriched in the T2_3h samples (Fig. 2D). DEGs in the T1_48h samples enriched GO terms related to translation, amide biosynthetic and metabolic process, and organonitrogen compound biosynthetic and metabolic process, whereas DEGs in the T2_48h samples enriched GO terms related to drug response and transport, and nitrogen compound transport. Taken together, despite a partial overlap in stress response, there were distinct transcriptional responses to recurrent salt stress in maize.

### Identification of salt stress memory genes in maize

**Fig. 3:**
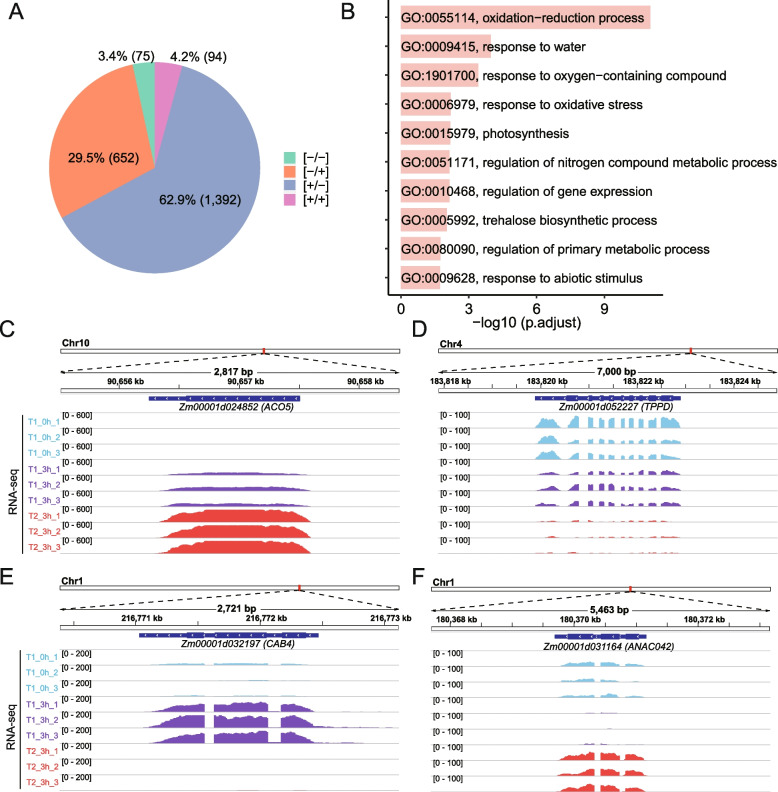
Analysis of salt stress memory genes in maize. **A** Proportion of different types of salt stress memory genes. Genes that are up-regulated in both T1_3h vs T1_0h and T2_3h vs T1_3h are designated as [+ / +], genes that are down-regulated in both comparisons are designated as [-/-], genes that are up-regulated in T1_3h vs T1_0h but down-regulated in T2_3h vs T1_3h or vice versa are designated as [+/-] or [-/ +] genes, respectively. **B** GO enrichment analyses of salt stress memory genes. The selected 10 enriched GO biological processes are shown. **C**-**F** The RNA-seq tracks for representative salt stress memory genes

To explore the mechanisms underlying salt memory in maize, we identified salt stress memory genes (SMGs). Stress memory genes refer to genes whose transcriptional responses significantly differ between the first and subsequent stress events, which can be categorized into four types [7, 11, 16]. Accordingly, SMGs were identified by conducting a comparative analysis of DEGs between T1_3h vs. T1_0h and T2_3h vs. T1_3h, as previously done in switchgrass and rice [7, 16]. Genes displaying an up-regulation in both T1_3h vs T1_0h and T2_3h vs T1_3h ([+ / +]), down-regulation in both comparisons ([-/-]), or up-regulation in T1_3h vs T1_0h but down-regulation in T2_3h vs T1_3h or vice versa ([+/-] or [-/ +]) were considered to be SMGs. A total of 2,213 SMGs were identified, consisting of 94 [+ / +] genes, 75 [-/-] genes, 1,392 [+/-] genes, and 652 [-/ +] genes (Fig. 3A, Table S4). Notably, the number of [+ / −] genes was higher than the other three types of genes, accounting for 62.9% of all SMGs.

Then, we performed GO enrichment analysis on these SMGs, unveiling their significant enrichment in 30 pathways (adjusted *P*-value ≤ 0.05) (Table S5). The representative enriched pathways are involved in oxidation–reduction process, photosynthesis, regulation of gene expression, and trehalose biosynthetic process (Fig. 3B). For example, the *Zm00001d024852* (*ACO5*) gene, which plays a role in the oxidation of 1-aminocyclopropane-1-carboxylic acid (ACC) to ethylene [24], exhibited an up-regulation in both T1_3h vs T1_0h and T2_3h vs T1_3h ([+ / +]) (Fig. 3C). In contrast, the *Zm00001d052227* (*TPPD*) gene, which converts trehalose-6-phosphate (T6P) to trehalose [25], displayed a down-regulation in both comparisons ([-/-]) (Fig. 3D). The gene related to the light reactions of photosynthesis, *Zm00001d032197* (*CAB4*) [26, 27], was up-regulated in T1_3h vs T1_0h but down-regulated in T2_3h vs T1_3h ([+/-]) (Fig. 3E). However, the *Zm00001d031164* (*ANAC042*) gene, which encodes a transcription factor [28], was down-regulated in T1_3h vs T1_0h but up-regulated in T2_3h vs T1_3h ([-/ +]) (Fig. 3F).

### Transcription factors network analysis for identification of crucial regulators

**Fig. 4:**
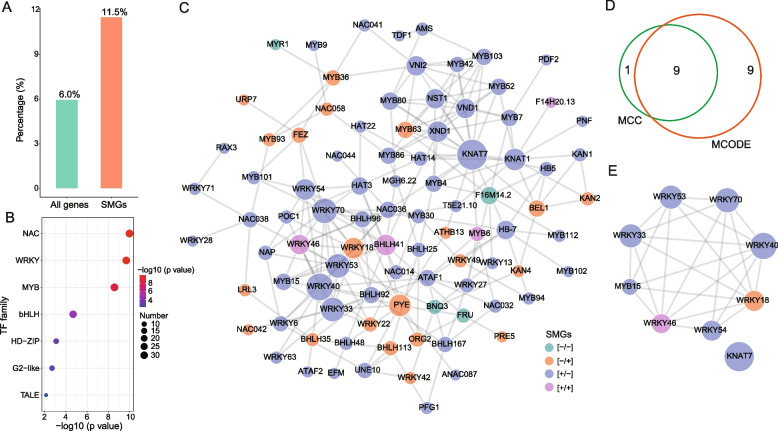
Identification of transcription factors and PPI network analysis. **A** Proportion of TF genes in the SMGs. All genes in the genome are the control group. **B** The bubble diagram showing the significantly enriched TF families in the SMGs. The bubble size represents the number of SMGs in that family, and the color represents the P value of enrichment analysis. All genes in the genome are the control group. Fisher’s exact test was used to identify significantly enriched TF families. **C** PPI network of selected TFs. The network was constructed and visualized using STRING and Cytoscape, respectively. The node size represents the degree of connectivity, with larger sizes indicating higher connectivity. The node color represents the types of SMGs. **D** Venn diagram showing the overlap of hub TFs identified using the MCODE and MCC analyses. **E** The PPI network of hub TFs

Given the important role of TFs in the formation and maintenance of memory behavior in both animals and plants [29–33], we wonder whether TFs participated in salt stress memory in maize. By comparing sequence homologies with entries in the PlantTFDB database, 11.5% (255/2,213) of SMGs were identified as TF genes distributed across 38 families. Notably, the NAC (33 genes) and MYB (33 genes) families exhibited the highest abundance, closely followed by the WRKY (31 genes) and bHLH (29 genes) families (Table S6). Interestingly, the proportion of TF genes in SMGs was significantly higher than that in the maize genome (6.0% of all genes [2,361/39,498] were TF genes; *P* < 0.01, Fisher’s exact test) (Fig. 4A). Additionally, we assessed the enrichment of each TF family within SMGs and found that seven TF families, including NAC, WRKY, MYB, bHLH, HD-ZIP, G2-like, and TALE, exhibited marked enrichment (*P* < 0.01, Fisher’s exact test) (Fig. 4B). Altogether, these results indicate that specific TFs are involved in the regulation of salt stress memory in maize.

To further reveal potential relationships among the enriched TFs, we established a protein–protein interaction (PPI) network using the STRING database. As shown in Fig. 4C, the PPI network consisted of 92 nodes (TFs) and 219 edges (interactions). Subsequently, we identified hub TFs within the network using the Molecular Complex Detection (MCODE) and Maximal Clique Centrality (MCC) algorithms, yielding 18 and 10 TFs, respectively. Notably, nine TFs, namely WRKY40, WRKY46, WRKY53, WRKY18, WRKY33, WRKY70, MYB15, KNAT7, and WRKY54, were considered crucial hub TFs due to their identification by both methods (Fig. 4D-E). Interestingly, except for WRKY18 and WRKY46, the remaining seven TFs belong to [+ / −] memory genes. Several of these TFs have been demonstrated to play an important role in the regulation of salt stress responses. For example, WRKY46 regulates lateral root development under salt stress conditions by regulating ABA signaling and auxin homeostasis [34, 35]. Furthermore, WRKY33 [36, 37], WRKY40 [38], WRKY53 [39], and MYB15 [40] act as transcriptional activators enhancing salt tolerance, whereas WRKY18 heightens sensitivity to salt and osmotic stress in plants [41].

### Validation of expression pattern of TF genes using qRT-PCR

**Fig. 5:**
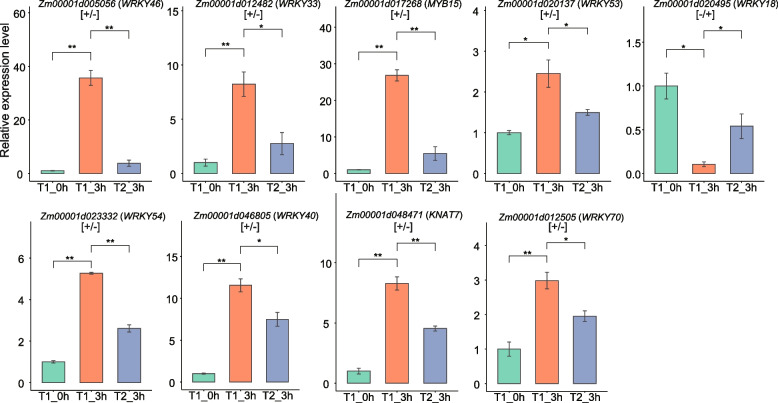
Quantitative real-time PCR analysis of crucial TF genes. The *EF1*-α gene was amplified as an internal control. The expression level of indicated genes in the T1_0h was set to 1. Data are presented as the mean ± standard error (SE) (*n* = 3). Student’s t-test was used for statistical analysis (* *P* < 0.05, ** *P* < 0.01)

To validate the results of RNA-seq, we conducted quantitative real-time PCR (qRT-PCR) experiments to quantify the expression levels of the nine crucial TF genes (Fig. 5, Figs. S1 and S2, Table S7). The measured expression profiles of these genes were consistent with the results obtained from the RNA-seq analysis, with the exception of *Zm00001d005056*. *Zm00001d005056* was identified as a [+ / +] gene via RNA-seq but as a [+ / −] gene via qRT-PCR. This discrepancy may be attributed to technical or biological reasons [42]. Overall, the RNA-seq data demonstrated high reliability in assessing gene expression in response to salt stress in maize. The nine TFs identified could potentially contribute to the improvement in salt tolerance during recurrent exposure to salt stress.

### Predicted crucial TFs directly regulated SMGs

**Fig. 6:**
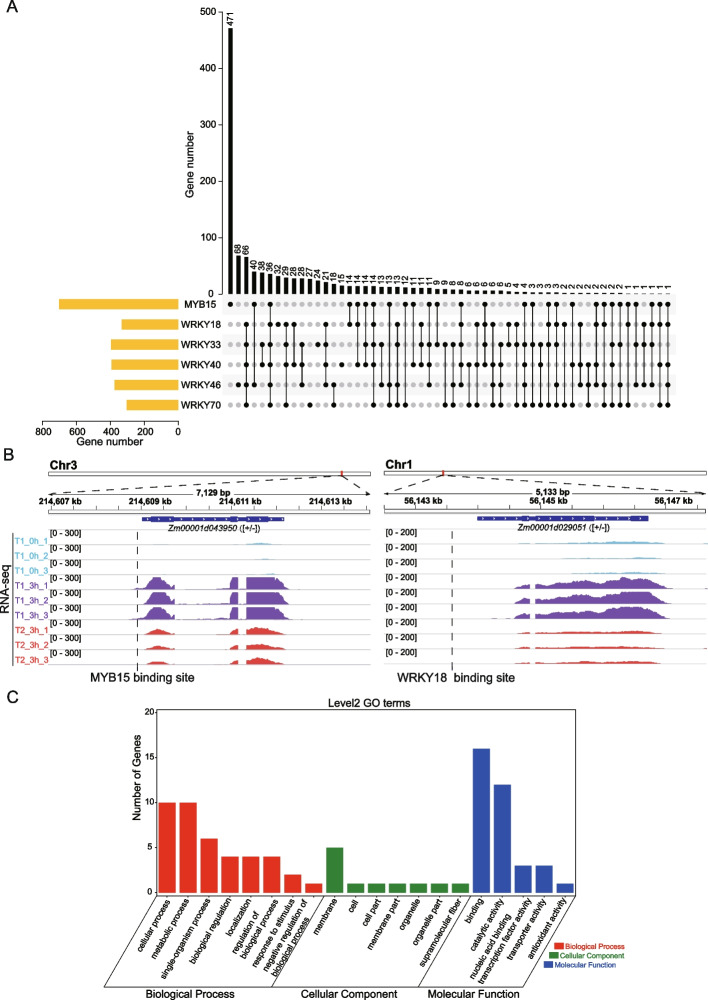
Predicted binding sites and target genes of crucial TFs. **A** Upset diagram showing the overlap of predicted target genes of WRKY40, WRKY46, WRKY18, WRKY33, WRKY70, and MYB15. **B** Representative salt stress memory genes with putative TF binding at promoters. **C** GO analysis of the common targets of crucial TFs

We want to know whether the nine crucial TFs identified have the capacity to directly regulate SMGs. To this end, we scanned the promoter regions (defined as 1 kb upstream of the transcription start site [TSS] of a gene) of SMGs using the corresponding TF binding motifs to infer potential TF binding sites, and thus identified the target genes for each TF. Since only six of the nine TFs had accessible motifs in public databases, we concentrated our analysis on these six TFs, namely, WRKY40, WRKY46, WRKY18, WRKY33, WRKY70, and MYB15. We discovered 393 predicted target genes for WRKY40, 376 for WRKY46, 332 for WRKY18, 396 for WRKY33, 304 for WRKY70, and 701 for MYB15, respectively (Fig. 6A). Representative examples are shown in Fig. 6B. In total, 1,192 target genes were detected, accounting for more than half (53.9%) of all SMGs. Thus, these TFs tend to directly regulate the expression of SMGs. Interestingly, our analysis revealed that out of the 1,192 target genes, 36 were predicted to be the common targets for all six TFs (Fig. 6A), consisting of one [+ / +] gene, two [-/-] genes, 21 [+/-] genes, and 12 [-/ +] genes. We performed GO analysis on this group of genes, revealing their distribution across diverse categories (Fig. 6C). Within these categories, cellular process (27.8%, 10/36) and metabolic process (27.8%, 10/36), membrane (13.9%, 5/36), and binding (44.4%, 16/36) were prominently represented in biological process, cellular component and molecular function, respectively.

## Discussion

Plants subjected to abiotic stress can acquire stress memory, enhancing their responsiveness to subsequent stress events. Despite its importance, the molecular mechanisms driving this process remain unclear. To gain insights into this, we conducted experiments involving cycles of salt stress and restoration treatments in maize.

First, to assess salt stress memory in maize, we examined proline and MDA contents and SOD and POD activities, which are common indicators of plant stress tolerance [43–45]. Elevated SOD and POD activities, along with higher proline content and lower MDA content, are indicative of enhanced stress tolerance. Within the same duration of salt treatment, we observed higher proline content, SOD, and POD activities in T2 plants (those that were exposed to a second round of salt stress) than in T1 plants (those that were first exposed to salt stress). Conversely, MDA content was lower in T2 plants than in T1 plants (Fig. 1C), suggesting an enhanced adaptive response to salt stress during the second salt stress event. These memory-associated changes are similar to what has been observed in other plants [46, 47], indicating the presence of a physiological memory effect in maize following the first stress event.

In addition to physiological changes, we also observed differences in gene expression profiles between plants with recurrent exposure to salt stress and those encountering salt stress for the first time (Fig. 2). Consistent with results reported in coffee and rice [15, 16], we observed a reduced number of DEGs in maize plants previously exposed to salt stress. Further functional analysis revealed several GO terms enriched in both the first and second salt stress responses, including the oxidation − reduction process, response to oxidative stress, trehalose biosynthetic process, and cellular ion homeostasis. These enriched GO terms have been demonstrated to play a crucial role in enhancing stress tolerance in plants [48–50], indicating their importance across different stress events. In addition, the stress resistance response might reduce photosynthesis efficiency, impacting short-term plant productivity, while heightening tolerance to subsequent stress events and promoting long-term productivity gains [47, 51]. This phenomenon was verified in the present study as we observed a perturbation in photosynthesis only during the first salt stress event (3h) that recovered during subsequent stress events (Fig. 2D). Furthermore, we observed significant differences in the response processes during the second salt stress compared to the first. These contrasting responses demonstrate the presence of transcriptional memory of salt stress exposure in maize.

Genes associated with stress memory have been identified through transcriptomic analysis in several plants [7, 11, 13, 16]. These genes are distinguished by their capacity to respond to the initial stress event and subsequently adjust their expression during repeated exposure to the same stress, enabling the plant to refine its responses to recurrent stress events [16]. For example, in *Arabidopsis*, over 2,000 genes have exhibited memory responses [11, 47, 52]. In this study, we employed RNA-seq to identify genes related to salt stress memory in maize, resulting in the identification of 2,213 SMGs that displayed distinct expression profiles in subsequent stress events compared to the initial event (Fig. 3A). Among these SMGs, the most prominent group comprised genes with high expression levels during the first stress event but reduced expression during the second stress event or vice versa ([+ / −] or [− / +]). This finding is consistent with that of previous studies on *Arabidopsis*, switchgrass, and rice [7, 16]. Genes displaying [+ / −] or [− / +] response patterns have been classified as “revised response” genes because they “revise” their initial behavior following subsequent exposure to the same stress [11, 13, 16]. This underscores the pivotal role of revised response in the plant stress memory mechanism.

TFs have been proposed as pivotal factors in determining memory behavior [53–55]. In this study, we observed that 11.5% of the identified SMGs in maize belong to the TF category (Fig. 4A), indicating the involvement of TFs in the establishment and orchestration of salt stress memory in maize. Analysis of the PPI network suggested that a complex regulatory network was responsible for inducing stress memory. Notably, we obtained nine hub TFs within the network (Fig. 4D), which act as robust candidates for salt memory factors that activate protective genes in response to subsequent salt stress. Interestingly, seven out of these nine TFs were from the WRKY family, which are known to play important roles in plant responses to various biotic and abiotic stresses [56–58]. Despite the relatively limited knowledge about their roles in salt stress memory, six of these TFs, namely, WRKY18 [41], WRKY33 [36, 37], WRKY40 [38], WRKY46 [34, 35], WRKY53 [39], and MYB15 [40], have been demonstrated to be involved in the response to salt stress. Therefore, further functional studies, particularly with regard to transcriptional memory, are warranted. Furthermore, we found that over 53% of all identified SMGs can be directly regulated by the nine identified TFs (Fig. 6A), providing evidence of their active involvement in the processes of salt stress memory in maize. However, there were differences in response patterns, such as MYB15 exhibited [+/-] expression, whereas WRKY18 exhibited [-/ +] expression. Together, our results indicate that salt stress memory can be established in maize and plants pre-exposed to salt stress exhibit better resistance to subsequent salt stresses.

## Conclusions

Our study unveiled salt stress memory in maize, identifying 2,213 salt stress memory genes (SMGs) grouped into four distinct subcategories with altered responses to repeated stress. The most notable SMG groups exhibited revised responses to repeated stress, highlighting the significance of revised responses in plant stress memory. Moreover, we identified nine pivotal transcription factors (TFs) that govern these SMGs, offering potential genetic targets for enhancing salt stress resilience and crop productivity.

## Materials and methods

### Plant materials and growth conditions

Seeds of the maize inbred line B73 were germinated and grown in a greenhouse (13/11 h light/dark, 28/26 °C light/dark, and 50% relative humidity). Zhiying Zhu and Jinlei Han undertook the formal identification of the plant material used in the study, and no voucher specimens were collected. After the maize seedlings grew to the two leaves and one heart stage, they were transplanted and hydroponically cultivated in 1/2X Hoagland nutrient solution for five days. Thereafter, the plants were treated with 200 mM NaCl for 3 h (this corresponded to the first salt stress event, T1_3h). Non-stressed plants were simultaneously sampled as controls (T1_0h). Subsequently, half of the salt-stressed plants were incubated with 200 mM NaCl for another 45 h (a total exposure of 48 h, T1_48h). The remaining plants were transferred to 1/2X Hoagland nutrient solution for three days of recovery culture and subsequently exposed to salt stress (200 mM NaCl) for 3 h and 48 h (the second salt stress event, T2_3h and T2_48h) (Fig. 1A). Root tissues from both control and salt-treated plants were collected for further analysis.

### Measurement of physiological parameters

Physiological parameters were measured using corresponding assay kits according to the manufacturer’s protocols. Specifically, the Proline Content Assay Kit (Solarbio, Cat#BC0290) was employed to quantify proline content using an acidic ninhydrin-based method. The absorbance was detected at a wavelength of 520 nm with a spectrophotometer. The Malondialdehyde (MDA) Content Assay Kit (Solarbio, Cat#BC0020) was utilized to measure MDA content, employing the thiobarbituric acid (TBA)-based colorimetric method, with absorbance measured at 532 nm. Superoxide Dismutase (SOD) activity was determined using a nitroblue tetrazolium colorimetric SOD Activity Detection Kit (Solarbio, Cat#BC0170), with absorbance measured at 560 nm. Peroxidase (POD) activity was assessed through the guaiacol method with the colorimetric POD Activity Detection Kit (Solarbio, Cat#BC0090), and absorbance measurements were conducted at 470 nm. All experiments were performed in triplicate.

### RNA-seq and data analysis

Total RNA was extracted from root samples using the Omega Plant RNA kit (Omega Bio-tek, Cat. no. R6827-01) and used to make RNA-seq libraries with the Illumina TruSeq RNA Kit (NEB, Cat. no. E7530) following the manufacturer's instructions. The libraries were sequenced with the Illumina NovaSeq 6000 platform in the 150 bp paired-end mode. Three biological replicates were performed for the RNA-seq experiment. Sequencing raw reads were quality filtered and trimmed by using the Trim_Galore v.0.6.7 package (https://www.bioinformatics.babraham.ac.uk/projects/trim_galore/). Generated clean reads were then mapped to the maize reference genome using TopHat2 v.2.1.1 [59] with default settings. The genome sequence and annotation files for maize (RefGen_V4) were downloaded from Phytozome (https://phytozome-next.jgi.doe.gov). Cufflinks v.2.2.1 [60] was employed to calculate the gene expression level represented by FPKM (fragments per kilobase of transcript per million mapped reads) values. To identify differentially expressed genes (DEGs), featureCounts v.2.0.1 [61] was used to calculate the read counts for each gene, and differential expression analysis was performed using DESeq2 [62]. Genes with an adjusted *p*-value < 0.05 and |log2(fold change)|> 1 were considered differentially expressed. Gene Ontology (GO) enrichment analysis was performed using the Omicshare online tools (www.omicshare.com/tools).

To visualize the mapped reads, the BAM format files were converted to the bigwig format using the bamCoverage function in deepTools v.3.1.3 [63] with a bin size of 10 bp and RPKM normalization. Genome browser images were made using the Integrative Genomics Viewer (IGV) (https://software.broadinstitute.org/software/igv/) with bigwig files processed as described above.

### Identification of salt stress memory genes

Salt stress memory genes (SMGs) were identified as described previously [7]. Briefly, DEGs between the T1_3h vs T1_0h and T2_3h vs T1_3h were compared, and four types of genes were considered to be SMGs: (1) those up-regulated in both T1_3h vs T1_0h and T2_3h vs T1_3h ([+ / +]), (2) down-regulated in both comparisons ([-/-]), and (3) up-regulated in T1_3h vs T1_0h and down-regulated in T2_3h vs T1_3h ([+/-]) or (4) vice versa ([-/ +]).

### Protein–protein interaction network analysis

A protein–protein interaction (PPI) network was constructed based on the STRING database (https://string-db.org/) and visualized using Cytoscape v.3.9.1 [64]. To identify hub genes, Cytoscape cytohHubba [65] plugin with the Maximal Clique Centrality (MCC) method or the Molecular Complex Detection (MCODE) [66] plugin was utilized.

### Defining predicted binding sites for TFs

The TF DNA binding motifs were downloaded from the PlantTFDB database (http://planttfdb.gao-lab.org/). Potential TF binding sites were determined by scanning motif occurrences in the region of interest by using the FIMO v.5.4.1 [67] with default settings. Candidate target genes for a given TF were defined as those with the TF binding motif in the promoter region, which was defined as 1 kb upstream of the transcription start site (TSS) of the gene.

### Quantitative RT-PCR analysis

To generate a qRT-PCR template, the extracted RNA was reverse transcribed using the StarScript II First-strand cDNA Synthesis Mix With gDNA Remover (Genstar, Cat#A224-10). We performed qRT-PCR using 2X RealStar Green Fast Mixture (Genstar) on a CFX Connect Real-Time PCR Detection System (Bio-Rad). The 2^−ΔΔCT^ method [68] was utilized for normalizing and calculating relative expression levels. Each qRT-PCR assay was repeated three times with three independent RNA preparations, and the maize *EF1*-α gene (NM_001112117) was used as an internal control for normalization [69, 70]. The nucleotide sequences of primers are listed in Table S7.

### Supplementary Information

**Additional file 1: Table S1.** Sequencing and mapping statistics for the RNA-seq data.

**Additional file 2: Table S2.** Differentially expressed genes (DEGs) between the control and salt stressed samples.

**Additional file 3: Table S3.** GO enrichment in biological process of DEGs.

**Additional file 4: Table S4.** Salt stress memory genes (SMGs) identified in maize.

**Additional file 5: Table S5.** GO enrichment in biological process of SMGs.

**Additional file 6: Table S6.** Transcription factor involved in salt stress memory.

**Additional file 7: Table S7.**  List of the primers for qRT-PCR analysis of the genes.

**Additional file 8: Figure S1.** PCR Efficiency.

**Additional file 9: Figure S2.** Melting curves of 10 genes were obtained using CFX Manager (Bio-Rad).

## Data Availability

The RNA-seq data described in this work have been deposited to the Genome Sequence Archive (GSA) database (http://gsa.big.ac.cn/) under the accession number CRA010351.
