# Supplementary material for: Dynamic physiological and transcriptomic changes reveal memory effects of salt stress in maize
Source: BMC Genomics. 2023 Dec 1;24:726. doi: 10.1186/s12864-023-09845-w (PMC10690987; doi:10.1186/s12864-023-09845-w)
Supplement: Supplementary file 8 — Additional file 8: Figure S1. PCR Efficiency. [file 12864_2023_9845_MOESM8_ESM.pdf]

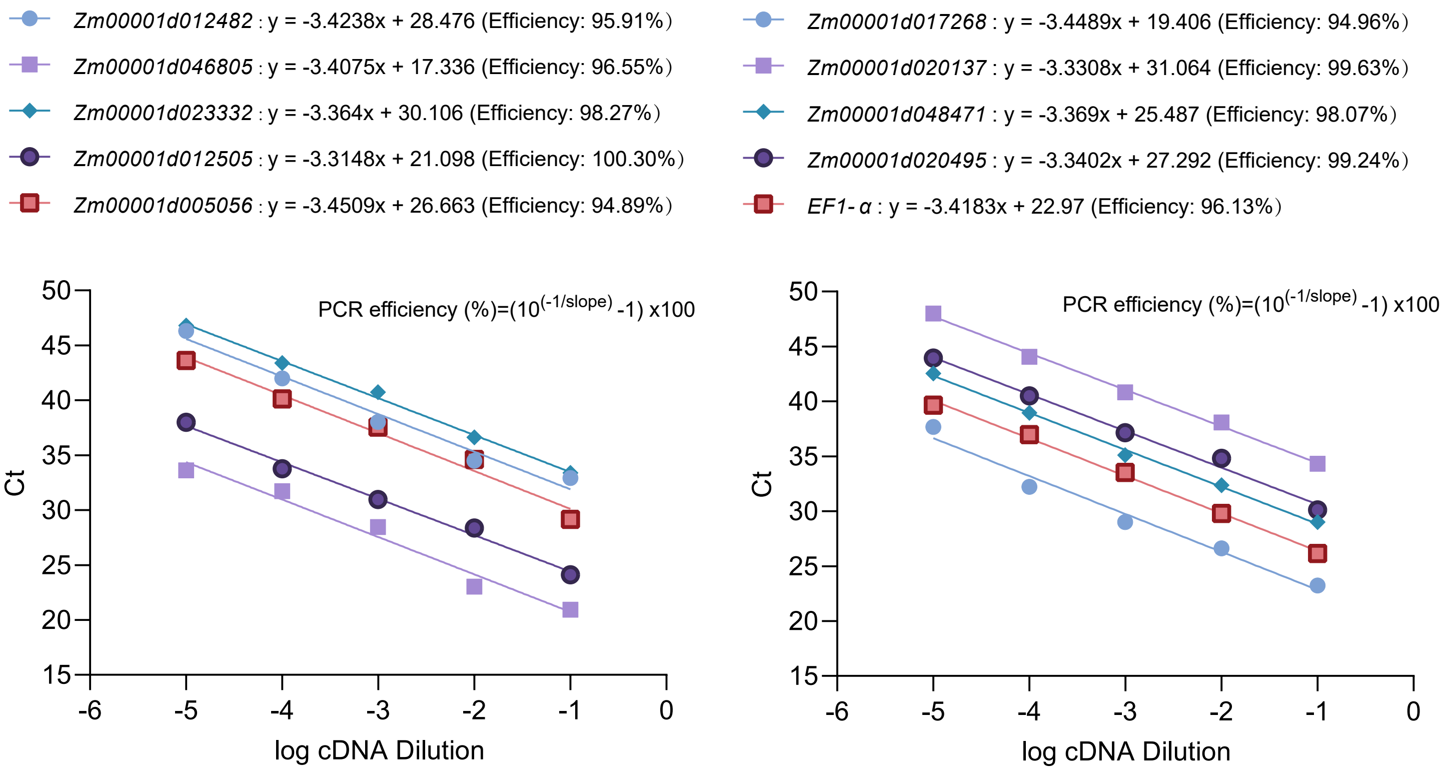

Figure S1. PCR Efficiency. PCR efficiency was determined for ten primer pairs using a five point 1:10 serial dilution of cDNA, which was synthesized by the reverse transcription of total RNA. After real time PCR, Ct values were plotted against the log cDNA dilution factor, and the slope of the plot was calculated. PCR efficiency was then determined using the equation, PCR efficiency (%) = (10 (-1/slope) - 1) *100, as described in Qiagen's Real-Time PCR Brochure (www.gene-quantification.de/qiagen-qpcr-sample-assay-tech-guide-2010.pdf).
