# Supplementary material for: Dynamic physiological and transcriptomic changes reveal memory effects of salt stress in maize
Source: BMC Genomics. 2023 Dec 1;24:726. doi: 10.1186/s12864-023-09845-w (PMC10690987; doi:10.1186/s12864-023-09845-w)
Supplement: Supplementary file 9 — Additional file 9: Figure S2. Melting curves of 10 genes were obtained using CFX Manager (Bio-Rad). [file 12864_2023_9845_MOESM9_ESM.pdf]

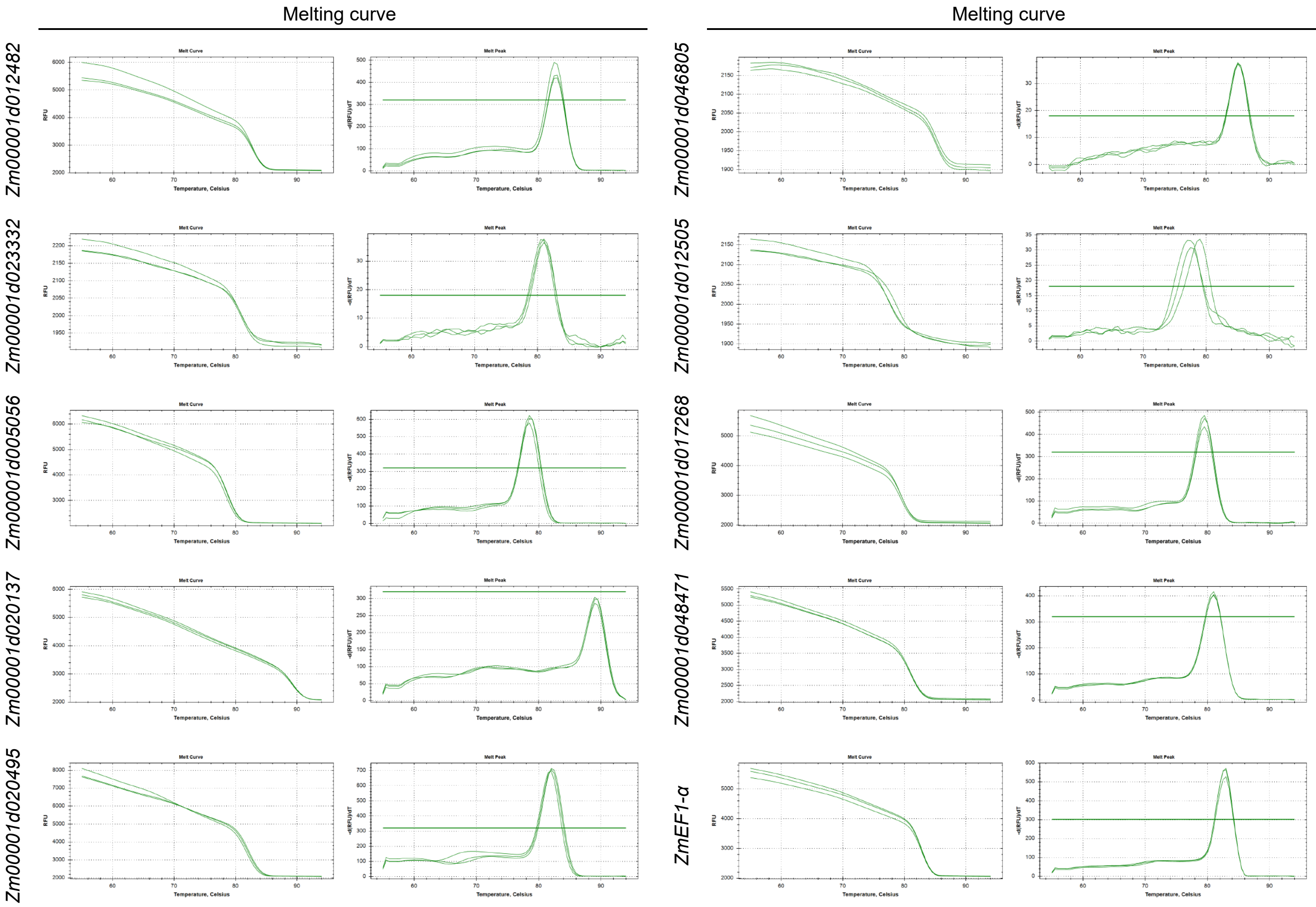

Figure S2. Melting curves of 10 genes were obtained using CFX Manager (Bio-Rad). Three different clusters show representative melting curves of three technical replicates using T1_0h sample as template. The left panel represents the original melting curve and the right panel represents the melting peaks, respectively. A single peak indicates a single qRT-PCR product. -d(RFU)/dT = Negative derivative (-d) of relative fluorescence units (RFU) over temperature (dT).
